# Dispositional need to belong and increased eating after social exclusion

**DOI:** 10.3389/fpsyg.2022.1095636

**Published:** 2023-01-12

**Authors:** Michihiro Kaneko, Yoshiyuki Ueda

**Affiliations:** ^1^Graduate School of Education, Kyoto University, Kyoto, Japan; ^2^Institute for the Future of Human Society, Kyoto University, Kyoto, Japan

**Keywords:** Cyberball game, dispositional need to belong, eating, emotion, social exclusion

## Abstract

Social exclusion affects the fundamental needs of individuals, and their coping behavior is moderated by their dispositional need to belong. Eating can be one such behavior. However, it is unclear how people with a higher or lower dispositional need to belong respond with respect to food consumption in response to social exclusion. Thus, this study aims to investigate which of these groups eat more food after social exclusion. Ninety-seven university students in Japan participated in two types of Cyberball games (where they either experienced social exclusion or social inclusion) in which their social exclusion status was manipulated. They subsequently participated in a test in which they ate as many cookies as they desired. Finally, they answered questions about their dispositional need to belong. Their BMI was also recorded. Results showed that when socially excluded, individuals with a low need to belong increased their consumption, while those with a high need to belong did not. We suggest that people with a lower need to belong are more inclined to focus on goals other than inclusion and instead use eating as a coping mechanism.

## 1. Introduction

Social exclusion has negative effects on humans, which can be observed through the development of adaptive responses (Williams, 2009). Experiencing social exclusion impairs fundamental needs, and encourages people to engage in coping behaviors to restore them (Williams, 2009; Hartgerink et al., 2015). Therefore, the effects of social exclusion are not only reflected in psychological and physiological reactions, but also in behavioral responses, such as eating (Baumeister et al., 2005^1^; Oaten et al., 2008; Senese et al., 2020). Moreover, it is known that the effect of social exclusion depends on individual differences, especially the dispositional need to belong (Koudenburg et al., 2013; Thau et al., 2015; Iannone et al., 2018; Pfundmair and Wetherell, 2019). The purpose of this study is to examine whether the dispositional need to belong moderates eating after social exclusion, to reveal coping mechanisms for social exclusion, and suggest the potential risks for binge eating and obesity.

### 1.1. Effects of social exclusion on fundamental needs and their individual differences

In previous studies, the fundamental needs impaired by social exclusion are usually listed as belonging, self-esteem, control, and a meaningful existence (Williams, 2009). To cope, excluded people tend to behave in a manner that satisfies their need to belong (Maner et al., 2007). The coping behaviors vary among individuals and across contexts (Hayman Jr et al., 2015). As social exclusion disconnects people from others, their dispositional need to belong to groups can be considered as one of the personality traits determining individual differences (Pickett et al., 2004; Leary et al., 2013; Beekman et al., 2016). So far, the moderation effect of the dispositional need to belong after social exclusion has been investigated in reconnection behaviors. For instance, individuals with a high dispositional need to belong tried to gain acceptance from the group when they were excluded (Thau et al., 2015; Iannone et al., 2018; Pfundmair and Wetherell, 2019). Furthermore, those with a low dispositional need to belong did not feel the need to abide by group opinions (Koudenburg et al., 2013).

However, fundamental needs are not restricted to these domains alone. The need for food is one fundamental need; exclusion from others is thought to have posed a risk of food acquisition failure in our evolutionary history (Spoor and Williams, 2007), and research shows that social exclusion can increase eating (Baumeister et al., 2005; Oaten et al., 2008; Senese et al., 2020). Although it has been revealed that people with a higher dispositional need to belong desired reconnection behavior after exclusion, it is unclear whether this expands to other types of coping behaviors, such as increased eating. Detecting people who eat more in response to social exclusion enables us to understand the mechanisms to help them cope with social exclusion by eating, and identify those with the potential risk of binge eating and obesity.

In this study, we hypothesized that the effect of social exclusion on food consumption depends on a person’s dispositional need to belong (Hypothesis 1: H1). To empirically investigate the causal relationship between social exclusion and eating, we employed a Cyberball game (Williams et al., 2000), ostensibly representing the mental visualization task. This game is a popular measure for manipulating social exclusion and its effect on eating (Oaten et al., 2008). Instead of measuring the subjective desire for food, we measured participants’ actual food intake through our study. Employing this procedure enabled us to investigate whether such a coping behavior moderated by a dispositional need to belong is actually observed as a behavior. We also had participants join both the exclusion and inclusion conditions, enabling us to interpret the result in terms of intra-individual changes rather than inter-individual differences.

Furthermore, for the moderation effect of the dispositional need to belong, we expect that individuals with a high dispositional need to belong are expected to increase their food intake when socially excluded, compared to when they are accepted; this amount is greater than the amount of food that individuals with a low dispositional need to belong eat when they are excluded, compared to when they are accepted (Hypothesis 2: H2). This is because a connection with others is important and required to the former individuals compared to those with a low dispositional need to belong (Koudenburg et al., 2013; Thau et al., 2015; Iannone et al., 2018; Pfundmair and Wetherell, 2019), and the former people must cope with their exclusion by desiring things that comfort them.

Further, we exploratorily investigated whether emotions were related to these processes: there was a possibility that the effect of social exclusion on emotions was moderated by individual differences in the dispositional need to belong and that emotions were related to eating. Although the impact of positive and negative emotions on eating has previously been posited (e.g., Macht, 2008), experimentally induced negative emotions are neither related to (Evers et al., 2018) nor mediate the influence of social exclusion on the amount of food consumed (Baumeister et al., 2005).

## 2. Materials and methods

### 2.1. Participants

We recruited 100 undergraduate and graduate students from a Japanese university as study participants. To detect the two-way interaction between social exclusion and dispositional need to belong, we conducted an *a-priori* power analysis with G*power 3.1 (Faul et al., 2007). We specified an error probability (i.e., α) of 0.05, a power (i.e., 1-β) of 0.80, and an effect size (i.e., f^2^) of 0.15 (i.e., middle size) with linear multiple regression: fixed model, R^2^ deviation from zero. Although the effect size of social exclusion is usually known to be large from the previous meta-analysis (Hedges’s g = 1 ~ 2: Hartgerink et al., 2015), we did not have any information on the dispositional need to belong regarding eating. Therefore, we decided that the predicted effect size was medium. The analysis results suggested a total recommendation sample size of 77. To pre-empt a lack of data due to technical reasons, we decided to recruit 100 participants. They were fluent in Japanese and aged ≥18 years old. Three participants were excluded: one withdrew after the first session; one ate less than 1 g both in the first and second sessions; and one refused to measure height and weight to calculate BMI. Data from 97 participants were analyzed. Participants visited the laboratory twice, experiencing both exclusion and inclusion conditions on separate days. They received JPY 3000 (approximately USD 30) as compensation.

Participants were informed that they could withdraw at any time, about the possible risks associated with participation, and were assured of the confidentiality and anonymity of their data. All participants provided written informed consent.

The research protocol was approved by the ethics committee of Kyoto University before the study was conducted (approval number: CPE308). The study was conducted in accordance with the guidelines stipulated by the Declaration of Helsinki. At the end of the second day, participants were debriefed, thanked for their services, and given their compensation. None of the participants reported realizing that the hypotheses that social exclusion made people consume more food and/or this effect was moderated by the dispositional need to belong.

#### 2.1.1. Demographics

Participants’ sex and age were recorded (54 men, 41 women, and 2 who did not want to report their sex; *M*_age_ = 19.94 years, *SD* = 2.16, Range = 18–27). Forty-seven participants were assigned to the inclusion condition on the first day and the exclusion condition on the second day (24 men, 22 women, and 1 who did not want to report their sex; *M*_age_ = 19.81 years, *SD* = 2.04, Range = 18–25) while the other 50 participants were assigned to the exclusion condition on the first day and the inclusion condition on the second day (30 men, 19 women, and 1 who did not want to report their sex; *M*_age_ = 20.06 years, *SD* = 2.28, Range = 18–27). These assignments were made randomly to ensure counterbalance across participants.^2^

### 2.2. Materials

#### 2.2.1. Emotional state

Using the Japanese version of the Positive and Negative Affect Schedule (PANAS; Watson et al., 1988; Kawahito et al., 2011), participants reported their current emotional states before and after social exclusion manipulation. Participants rated 10 positive and negative emotional adjectives (e.g., interested or irritable) on a 6-point Likert scale (1 = *not at all* to 6 = *extremely*). The PANAS score was calculated separately for positive and negative emotional states through averages.

#### 2.2.2. Social exclusion manipulation

Using the Cyberball game, the participants’ social exclusion status was manipulated. Each was informed that they would play with two others; however, the other players were manipulated by a computer program. Participants were asked to imagine the game vividly, as if they were playing in the real world. The game was programmed to end after the ball was tossed 50 times. Participants were randomly assigned to either the inclusion or exclusion conditions in the Cyberball game in the first experiment and to the other condition in the second. In the inclusion condition, participants had approximately a 33% ball possession (i.e., all players possessed the ball equally), while they had only a 12% ball possession (i.e., 6 out of 50 times) in the exclusion condition, initially receiving the ball infrequently and not receiving it at all in the latter half of the game. They were informed that their opponents in the first and second experiments were different, and opponents’ displayed initials and birthdays (e.g., Rt0627) changed.

#### 2.2.3. Bogus taste test

Using a bogus taste test (Robinson et al., 2017), the volume of food consumed was measured. In the test, participants were instructed to participate in a taste and smell measurement. Previous studies have validated the use of the bogus taste test (Robinson et al., 2017). It has also been used in a previous study on the effect of social exclusion on the volume of food consumed (Oaten et al., 2008). In our study, participants were offered a bowl containing small round cookies (*egg bolo*). Egg bolo is popular among children in Japan and most Japanese people have eaten it. We chose a small individual portion size per cookie (approximately 0.5 g) to detect even minor differences in consumption. We used two flavors (pumpkin and spinach); the order of flavor between the first and second experiments was random. The total weight of the cookies presented was approximately 120–125 g. The caloric values of the two flavors were similar (i.e., 382 kcal and 381 kcal/100 g for pumpkin and spinach, respectively). We calculated consumption by subtracting the post-weight from the pre-weight of the bowl containing the egg bolo. Participants were instructed to answer some questions, including taste and smell of the cookie eaten for the purpose of new measurement development after eating, and were informed that they could eat as much as they wanted for 3 min. They remained in place for 3 min, even if they had finished eating. None of the participants ate all the cookies that were offered. The weight of the remaining cookies was measured using a scale that could identify 0.1 g variances. After 3 min, participants answered a 12-item questionnaire about the cookies on a 6-point Likert scale (1 = *not at all* to 6 = *extremely*). Of the responses, we analyzed three items about preference for cookie taste, smell, and texture (we did not analyze other items because they were not related to cookie preferences).

#### 2.2.4. BMI

Participants’ height and weight were measured to calculate their BMI. BMI is calculated by weight divided by the square of height.

#### 2.2.5. Dispositional need to belong

The dispositional need to belong was measured with a 10-item scale, translated into Japanese (e.g., “I try hard to not do things that will make other people avoid or reject me”; Kobayashi et al., 2006; Leary et al., 2013). Answers were rated on a 5-point Likert scale (1 = *not at all* to 5 = *extremely*). The score was calculated by averaging all items after reverse scoring.

### 2.3. Procedures

The experiment was conducted at the university. To ensure that participants were moderately hungry, we requested that they avoid consuming food and drinks (except for water) for at least 2 h prior to participation; all participants adhered to this instruction. They came to the laboratory twice, at least 1 week apart, to participate in both the exclusion and inclusion conditions of social exclusion manipulation. The commencement time for each session was the same for all participants.

After arriving at the laboratory, participants were informed by the researchers that they would participate in two independent studies. To mask the true purpose of the social exclusion manipulation and the measurement of consumption, the first and second studies were represented as a computerized mental visualization exercise and an exercise in measuring taste and smell, respectively.

Then, they reported their current emotional state (Time 1), played the Cyberball game, and reported their current emotional state again (Time 2). Following that, they took part in a bogus taste test in which they ate food for a duration of 3 min and evaluated its taste, smell, and texture. After the above procedure on the second day, they were measured for their height and weight to calculate BMI, and reported on their dispositional need to belong and demographic variables.

### 2.4. Analyses

Although our sample size calculation was based on a between-participants design, before analyzing the data, we decided to change the analysis to a within-participants design using mixed-effects modeling because participants joined both the exclusion and inclusion conditions. In this study, we entered social exclusion status within-participant factors and dispositional need to belong between-participant factors into the mixed-effects model. In the analyses with mixed-effects modeling, we controlled age, day of the experiment (first vs. second), and BMI.^3^ This is because BMI is related to eating rate (Ohkuma et al., 2015), and eating rate is related to the amount of food consumed (Robinson et al., 2014). We standardized continuous variables, such as the dispositional need to belong, age, and BMI, and dummy coded discrete variables, such as the social exclusion status (i.e., exclusion condition = −0.5, inclusion condition = 0.5) and day of the experiment (i.e., first = −0.5, second = 0.5). We also entered participants’ ID as a random variable for intercepts.

## 3. Results

As eating showed higher skewness and kurtosis, we calculated the consumption (ln) score with a natural log transformation. The means of natural log transformed food consumption for the inclusion condition was 2.46 (*SD* = 0.62, Range = 0.64–4.00) and 2.48 for the exclusion (*SD* = 0.59, Range = 1.16–3.85). The descriptive statistics for the need to belong, BMI, consumption, and PANAS score for both inclusion and exclusion conditions are shown in Supplementary Table S1.

### 3.1. Manipulation check

For the social exclusion manipulation check, we first calculated positive and negative emotion change scores by subtracting Time 1 from Time 2, separately. Then, we employed a mixed-effects model with social exclusion status, the need to belong, and their interaction as independent variables, and a positive/negative emotion change score as the dependent variable, while controlling for Time 1 positive/negative emotion in addition to the control variables listed above. The analyses showed only a primary effect of social exclusion status for both positive and negative emotions (for positive emotion: *b* = −0.26, *SE* = 0.06, *p* < 0.001, 95% Confidence Interval (95% CI) = [−0.38, −0.14]; for negative emotion: *b* = 0.42, *SE* = 0.06, *p* < 0.001, 95% CI = [0.31, 0.54], respectively), but not for dispositional need to belong (positive: *b* = −0.00, *SE* = 0.04, *p* = 0.900, 95% CI = [−0.08, 0.07]; negative: *b* = 0.05, *SE* = 0.04, *p* = 0.262, 95% CI = [−0.03, 0.13]) and the interaction effects (positive: *b* = −0.06, *SE* = 0.06, *p* = 0.279, 95% CI = [−0.18, 0.05]; negative: *b* = 0.11, *SE* = 0.06, *p* = 0.060, 95% CI = [−0.01, 0.23]). This suggests that social exclusion decreases positive emotions and increases negative emotions, regardless of one’s degree of the dispositional need to belong (Supplementary Table S2).

To check whether egg bolo was preferred by participants, we tested whether preference for cookie taste, smell, and texture was higher than the theoretical mid-point of the scale (i.e., 3.5). The results of a one-sample *t*-test revealed that participants preferred the taste, smell, and texture of egg bolo [taste: *M* = 4.94, *t* (193) = 20.03, *p* < 0.001; smell: *M* = 4.19, *t* (193) = 7.47, *p* < 0.001; texture: *M* = 4.32, *t* (193) = 9.39, *p* < 0.001]. We also tested whether participants preferred one flavor over the other. The results of a paired *t*-test revealed that preferences did not differ in taste, smell, and texture of the egg bolo [taste: *t* (96) = 0.29, *p* = 0.771; smell: *t* (96) = 1.41, *p* = 0.161; texture: *t* (96) = 0.31, *p* = 0.758].

As dispositional need to belong was measured after the Cyberball game on the second day, we tested whether the social exclusion order affected reports of the dispositional need to belong by comparing the scores of participants who were socially excluded on the second day from those of participants who were socially included on the second day. The results of an independent two-sample *t*-test revealed that social exclusion order did not affect dispositional need to belong [*t* (95) = 0.38, *p* = 0.704].

### 3.2. The effect of social exclusion status and the need to belong on eating

**Figure 1:**
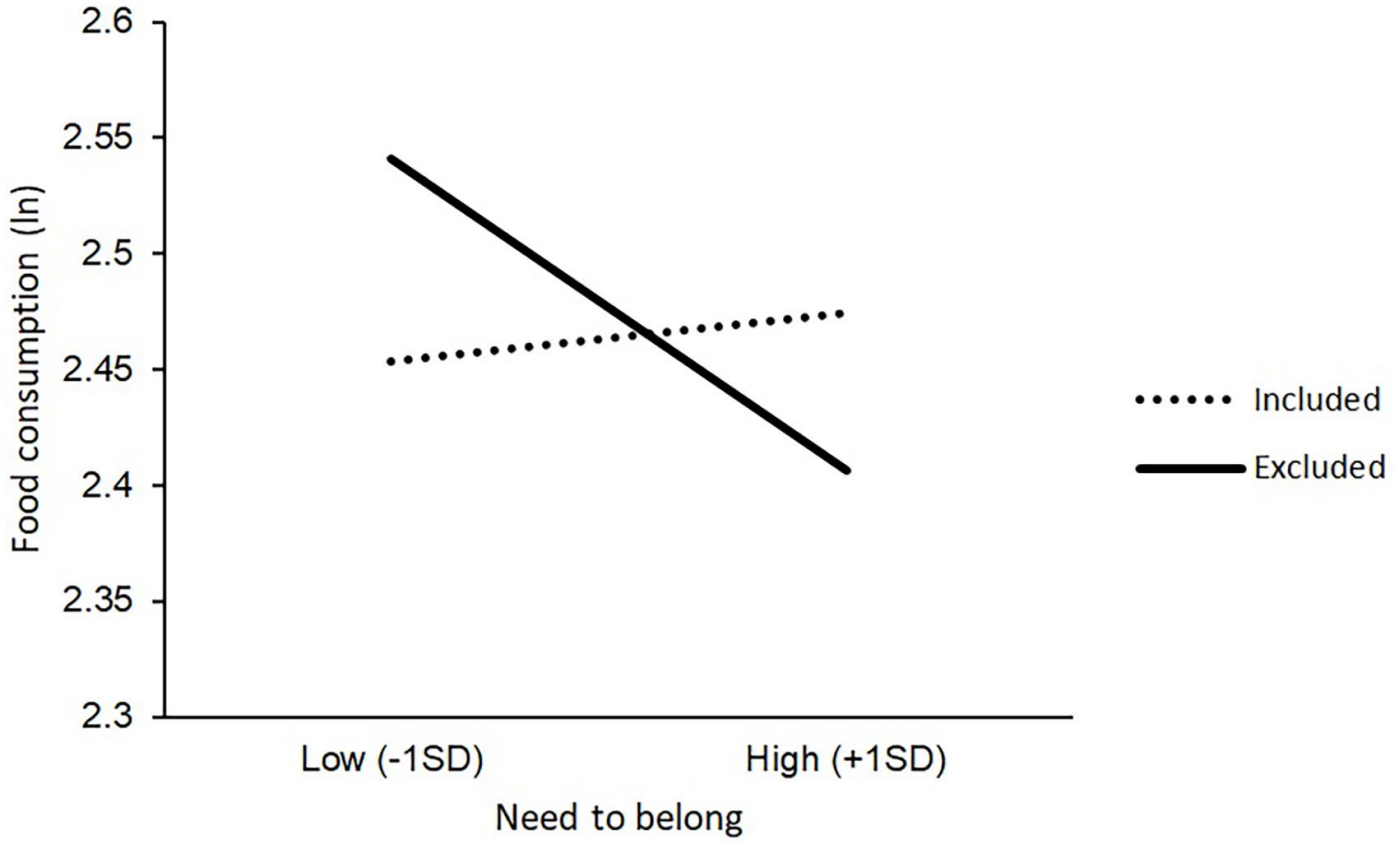
Interaction effect between social exclusion status and dispositional need to belong. *Note:* Food consumption is natural log transformed. The need to belong is standardized.

To determine whether the interaction between social exclusion and dispositional need to belong was related to eating, we employed a mixed-effects model with social exclusion status, the need to belong, and their interaction as independent variables, and consumption of food as the dependent variable. As shown in Table 1, the interaction was significant, indicating that H1 was supported. Subsequently, we compared consumption under the inclusion and exclusion conditions among participants with lower and higher needs to belong, using the simple slope analyses (Aiken et al., 1991). For this, we calculated the simple slope for the lower score of the need to belong by adding 1 *SD* to each participant’s need to belong score, and that of the higher need to belong score by subtracting 1 *SD* from each participant’s score.^4^ We found a significant increase in consumption in the exclusion condition compared with the inclusion condition, among those with a lower need to belong (−1 *SD*; *b* = 0.09, *SE* = 0.04, *p* = 0.026, 95% CI [0.01, 0.16]). However, we did not find a significant change among the participants with a higher need to belong (+1 *SD*; *b* = −0.07, *SE* = 0.04, *p* = 0.078, 95% CI [−0.14, 0.00]; Figure 1; Supplementary Table S3). The results revealed that the participants with a low dispositional need to belong ate more than those with a high dispositional need to belong when socially excluded. This indicated that H2 was not supported; rather, the results obtained trended in the opposite direction than predicted.

**Table 1:** The effect of social exclusion and dispositional need to belong on eating with a mixed-effects model.

Variable	*b*	*SE*	*t*	*p*	95% CI	
					LL	UL
Social exclusion status	0.01	0.03	0.35	0.731	−0.05	0.06
NTB	−0.03	0.06	0.51	0.611	−0.14	0.08
Social exclusion status × NTB	−0.08	0.03	2.85	0.005	−0.13	−0.02
Day	0.39	0.03	14.26	<0.001	0.33	0.44
Age	0.00	0.06	0.01	0.994	−0.12	0.11
BMI	0.10	0.06	1.71	0.091	−0.01	0.21

The dependent variable is the volume of food consumed with natural log transformation. Social exclusion status and day were dummy coded as − 0.5 and 0.5 for inclusion and exclusion, respectively; −0.5 for the first day and 0.5 for the second day. The NTB, age, and BMI were standardized. NTB, dispositional need to belong; BMI, body mass index; SE, standard error; CI, confidence interval; LL, lower limit of 95% CI; UL, upper limit of 95% CI.

### 3.3. Relationship between emotions and eating

We investigated whether emotions were related to food consumption. We used a mixed-effects model with a negative emotion change score as the independent variable, a negative emotion score before the Cyberball game (Time 1) as the control variable, and consumption as the dependent variable. The results indicated that negative changes in emotions were unrelated to the volume of food consumed (*b* = 0.01, *SE* = 0.04, *p* = 0.888, 95% CI = [−0.07, 0.08]; Supplementary Table S4).

We also investigated the effect of positive emotion change on food consumption with a mixed-effects model, using a positive emotion change score as the independent variable, a positive emotion score before the Cyberball game (Time 1) as the control variable, and consumption as the dependent variable. The results indicated that positive changes in emotions were unrelated to the volume of food consumed (*b* = −0.05, *SE* = 0.04, *p* = 0.287, 95% CI = [−0.13, 0.04]; Supplementary Table S5).

## 4. Discussion

This study investigated whether and how individuals’ dispositional need to belong moderates the effect of social exclusion on food consumption. Based on previous studies, we proposed two hypotheses: (H1) The dispositional need to belong moderates the effect of social exclusion on food consumption, and (H2) in response to social exclusion, individuals with a high dispositional need to belong eat more than those with a low dispositional need to belong. The results of the present study supported H1. This is the first empirical study showing that the effect of social exclusion on the volume of food consumed is moderated by the dispositional need to belong.

For H2, we found that contrary to the prediction, individuals with a low dispositional need to belong eat more than those with a high dispositional need to belong in response to social exclusion. The results may be interpreted to show that people with a low dispositional need to belong seek alternative resources (i.e., food) when they are not accepted by others. Considering the previous studies (Koudenburg et al., 2013; Thau et al., 2015; Iannone et al., 2018; Pfundmair and Wetherell, 2019), as reconnection with others is important for people with a higher dispositional need to belong but not for those with a low dispositional need to belong (Koudenburg et al., 2013), the latter may need to cope *via* other (i.e., non-reconnection) strategies, such as food consumption.

We also found that although negative emotions were increased and positive emotions decreased by social exclusion, this effect was not moderated by the dispositional need to belong. This corresponds to Williams (2009) observation that social exclusion is inevitably experienced negatively even if individuals’ dispositional need to belong is low. Moreover, we did not find that emotions were related to food consumption, as predicted by Macht (2008). The present results were consistent with a meta-analysis conducted by Evers et al. (2018), in which only certain groups of individuals (e.g., restrained eaters) tended to consume more in response to negative mood inductions.

Although the previous studies measured the effect of social exclusion on food consumption using a between-participants design (Baumeister et al., 2005; Oaten et al., 2008; Senese et al., 2020), the advantage of this study is that the same participants joined both the exclusion and inclusion conditions. It enabled us to observe the results of intra-individual changes rather than inter-individual differences. Thus, since each participant participated in the experiment for over 2 days, we used the effect of a day as a control variable. Therefore, we did not have any concrete hypotheses concerning this, but as shown in Table 1, the participants ate more on the second day than the first day. The overall amount of food consumption may have increased because they became accustomed to the experimental environments and relaxed on the second day.

The results can also be supported by the predictions of the goal-driven resource redistribution theory (Shilling and Brown, 2016). This theory proposes that social exclusion leads people to take different actions depending on their dispositional need to belong or long-term goals under limited cognitive resources. Coping strategies after social exclusion depend on the need for reconnection: people with a high dispositional need to belong allocate resources for reconnection, while those with a low dispositional need to belong allocate resources for other things. These variations in behaviors are considered to be evolutionarily adaptive (Shilling and Brown, 2016). From this perspective, in the present study, the reason why those with a high dispositional need to belong did not eat more might be attributed to their exclusive resource allocation for reconnection.

The neuroscience background regarding the increase in food consumption remains to be explored. Previous studies indicated that being ostracized (i.e., social exclusion) triggers the activation of the sympathetic nervous system (Kelly et al., 2012; Newman, 2014) and increased facial temperature (Paolini et al., 2016). In addition, higher facial temperature during social exclusion was associated with a decrease in happiness ratings and enhanced the need for social reconnection (Ponsi et al., 2019), which is in line with the social reconnection hypothesis (Maner et al., 2007). Therefore, based on the evidence that activation of the sympathetic nervous system inversely related to food intake (Bray, 1991), and the results of this study showing that people with a higher dispositional need to belong did not eat more when excluded than included, activation of the sympathetic nervous system after social exclusion may depend on their dispositional need to belong. People with a higher dispositional need to belong may activate the sympathetic nervous system more than those with a lower dispositional need to belong.

We must note further points. First, Oaten et al. (2008) showed that socially excluded individuals increased food consumption immediately after social exclusion. Furthermore, those with a higher fear of negative evaluations kept eating, even 45 min after social exclusion. Considering this, the dispositional need to belong moderated food consumption immediately after social exclusion, whereas fear of negative evaluation may modulate it sometime after social exclusion. Second, Pfundmair et al. (2015) suggest that the effects of social exclusion depend on individualistic vs. collectivistic cultures: After social exclusion, people in Germany reported a lower fulfillment of psychological needs (i.e., belonging, self-esteem, control, and meaningful existence) and showed higher heart rates than people in Turkey, India, Hong Kong and mainland China. Thus, people with a lower dispositional need to belong in individualistic cultures might eat more in response to social exclusion to complement their unfulfilled belonging than those in collectivistic cultures like Japan.

### 4.1. Limitations and future directions

Social exclusion resulted in increased eating among participants with a low dispositional need to belong. However, certain questions remain unanswered. First, a comparison between reconnection and eating in the same experiment is warranted. Exclusion is known to motivate those with a higher dispositional need to belong to set belonging goals (Koudenburg et al., 2013; Thau et al., 2015; Iannone et al., 2018; Pfundmair and Wetherell, 2019). Considering the results of the current study, it is not clear whether people with a low dispositional need to belong focus on eating only, or on reconnection as well. We should investigate this using a single paradigm.

Second, it is important to investigate whether food facilitates recovery from the negative effects of exclusion. In the present study, we did not specify the reason why people with a low dispositional need to belong increased their eating after social exclusion. One possible explanation is coping behavior. If it is actually due to coping behavior, then eating would mitigate the negative effects after social exclusion, including other fundamental needs or emotions.

Finally, it would be helpful to explore what type of need fulfillment is aimed at after social exclusion for people with a low dispositional need to belong. Although we found eating to be one such behavior, there are other types of fundamental needs, including self-esteem, control, and a meaningful existence (Williams, 2009). Moreover, there are other coping behaviors that fulfil fundamental needs; the desire for sleep, sex, or safety may be such needs (Maslow, 1970; Kenrick et al., 2010). Therefore, these need to be investigated in future research.

## 5. Conclusion

This study investigated whether the effect of social exclusion on coping behavior was moderated by an individual’s dispositional need to belong. We found that people with a low dispositional need to belong ate more small round cookies (i.e., egg bolo) after social exclusion compared to when they were included, while those with a high dispositional need to belong did not. However, the dispositional need to belong did not explain emotions after social exclusion, and emotions did not predict food consumption. Thus, food intake after social exclusion was modulated by a dispositional need to belong, not emotions, indicating that people with a low dispositional need to belong may be at greater risk of binge eating and obesity.

## Data availability statement

The datasets presented in this study can be found in online repositories. The names of the repository/repositories and accession number(s) can be found at: https://osf.io/enysm/?view_only=f8f0dc7aab7d40dbb9a7c6fcec3cec1e.

## Ethics statement

The studies involving human participants were reviewed and approved by the institutional ethics committee for experimental psychology at Graduate School of Education, Kyoto University (ethics number: CPE308). The participants provided their written informed consent to participate in this study.

## Author contributions

MK developed the study concept, contributed to the study design, and drafted the manuscript. MK performed testing, data collection, data analysis, and interpretation under the supervision of YU. YU provided critical revisions. All authors contributed to the article and approved the submitted version.

## Funding

This work was supported by JSPS KAKENHI [grant numbers JP16H01727, JP19J22920 and JP20H01786] and the INOUE ENRYO Memorial Grant, TOYO University 2021.

## Conflict of interest

The authors declare that the research was conducted in the absence of any commercial or financial relationships that could be construed as a potential conflict of interest.

## Publisher’s note

All claims expressed in this article are solely those of the authors and do not necessarily represent those of their affiliated organizations, or those of the publisher, the editors and the reviewers. Any product that may be evaluated in this article, or claim that may be made by its manufacturer, is not guaranteed or endorsed by the publisher.

## Footnotes

^1^Lurquin et al. (2016) reported that they failed to replicate the claim of Baumeister et al. (2005). They performed the conceptual replication concerning ego-depletion (i.e., self-control diminishes their resources and reduces the ability to it in subsequent behaviors), but not the direct replication of the effect of social exclusion. Increasing food consumption after social exclusion has been replicated in some studies (Oaten et al., 2008; Senese et al., 2020). Note that this might not be explained by lowered self-control, and should be considered as the involvement of potential moderator variables, as suggested by Lurquin et al. (2016).

^2^The experiment was conducted according to a counterbalance procedure. As a result, three participants were excluded from the analysis.

^3^The results were the same when BMI was not controlled.

^4^These calculations indicate that the dispositional need to belong score is set as 0 for participants whose need to belong is lower/higher by 1 *SD* than the average of participants, when calculating simple slope for the lower and higher need to belong, respectively.
