# Supplementary material for: Dispositional need to belong and increased eating after social exclusion
Source: Front Psychol. 2023 Jan 12;13:1095636. doi: 10.3389/fpsyg.2022.1095636 (PMC9878595; doi:10.3389/fpsyg.2022.1095636)
Supplement: Supplementary file 1 [file Table_1.DOCX]

Table S1

Descriptive statistics

|  |  |  | *M* |  | *SD* |  | Skewness |  | Kurtosis |  | α |
| --- | --- | --- | --- | --- | --- | --- | --- | --- | --- | --- | --- |
| NTB | |  | 3.19 |  | 0.60 |  | -0.22 |  | -0.27 |  | .80 |
| BMI | |  | 20.67 |  | 2.91 |  | 1.09 |  | 2.45 |  |  |
|  |  |  |  |  |  |  |  |  |  |  |  |
| Inclusion | |  |  |  |  |  |  |  |  |  |  |
|  | Food consumption |  | 14.13 |  | 9.53 |  | 1.82 |  | 3.94 |  |  |
|  | Food consumption (ln) |  | 2.46 |  | 0.62 |  | -0.06 |  | 0.18 |  |  |
|  | Positive emotion (Time 1) |  | 2.70 |  | 0.70 |  | -0.12 |  | -0.50 |  | .83 |
|  | Positive emotion (Time 2) |  | 2.64 |  | 0.73 |  | -0.03 |  | -0.82 |  | .83 |
|  | Negative emotion (Time 1) |  | 2.07 |  | 0.72 |  | 0.36 |  | -0.64 |  | .86 |
|  | Negative emotion (Time 2) |  | 1.86 |  | 0.66 |  | 0.43 |  | 0.08 |  | .88 |
|  |  |  |  |  |  |  |  |  |  |  |  |
| Exclusion | |  |  |  |  |  |  |  |  |  |  |
|  | Food consumption |  | 14.14 |  | 8.57 |  | 1.30 |  | 1.71 |  |  |
|  | Food consumption (ln) |  | 2.48 |  | 0.59 |  | -0.02 |  | -0.58 |  |  |
|  | Positive emotion (Time 1) |  | 2.71 |  | 0.71 |  | -0.31 |  | -0.48 |  | .83 |
|  | Positive emotion (Time 2) |  | 2.39 |  | 0.73 |  | 0.12 |  | -0.50 |  | .86 |
|  | Negative emotion (Time 1) |  | 2.02 |  | 0.71 |  | 0.43 |  | -0.42 |  | .85 |
|  | Negative emotion (Time 2) |  | 2.26 |  | 0.81 |  | 0.32 |  | -0.53 |  | .89 |

Note. NTB, dispositional need to belong; BMI, body mass index; *M*, mean; *SD*, standard deviation; α, Cronbach’s alpha.

Table S2

The effect of social exclusion status and dispositional need to belong on positive and negative emotion

| Dependent variable: Positive emotion change score | | | | | | | | | | | | |
| --- | --- | --- | --- | --- | --- | --- | --- | --- | --- | --- | --- | --- |
|  |  |  |  |  |  |  |  |  |  | 95% CI | | |
| Variable |  | *b* |  | *SE* |  | *t* |  | *p* |  | LL |  | UL |
| Social exclusion status |  | -0.26 |  | 0.06 |  | 4.52 |  | <.001 |  | -0.38 |  | -0.15 |
| NTB |  | -0.00 |  | 0.04 |  | 0.12 |  | .902 |  | -0.08 |  | 0.07 |
| Social exclusion status  × NTB |  | -0.06 |  | 0.06 |  | 1.10 |  | .274 |  | -0.18 |  | 0.05 |
| Positive emotion (Time 1) |  | -0.22 |  | 0.05 |  | 4.36 |  | <.001 |  | -0.32 |  | -0.12 |
| Day |  | 0.06 |  | 0.06 |  | 1.07 |  | .286 |  | -0.05 |  | 0.18 |
| Age |  | 0.04 |  | 0.04 |  | 1.03 |  | .308 |  | -0.04 |  | 0.12 |
| BMI |  | -0.01 |  | 0.04 |  | 0.17 |  | .869 |  | -0.08 |  | 0.07 |
|  |  |  |  |  |  |  |  |  |  |  |  |  |
| Dependent variable: Negative emotion change score | | | | | | | | | | | | |
|  |  |  |  |  |  |  |  |  |  | 95% CI | | |
| Variable |  | *b* |  | *SE* |  | *t* |  | *p* |  | LL |  | UL |
| Social exclusion status |  | 0.42 |  | 0.06 |  | 7.17 |  | <.001 |  | 0.31 |  | 0.54 |
| NTB |  | 0.05 |  | 0.04 |  | 1.13 |  | .262 |  | -0.04 |  | 0.13 |
| Social exclusion status  × NTB |  | 0.11 |  | 0.06 |  | 1.91 |  | .060 |  | -0.00 |  | 0.23 |
| Negative emotion (Time 1) |  | -0.36 |  | 0.06 |  | 6.34 |  | <.001 |  | -0.47 |  | -0.25 |
| Day |  | 0.01 |  | 0.06 |  | 0.16 |  | .875 |  | -0.11 |  | 0.12 |
| Age |  | -0.06 |  | 0.04 |  | 1.38 |  | .172 |  | -0.14 |  | 0.02 |
| BMI |  | -0.04 |  | 0.04 |  | 1.03 |  | .305 |  | -0.13 |  | 0.04 |

*Note*. Positive/negative emotion change score was calculated by subtracting Time 1 from Time 2 positive/negative emotion score. Social exclusion status and day were dummy coded as -0.5 and 0.5 for inclusion and exclusion, respectively; -0.5 for the 1^st^ day and 0.5 for the 2^nd^ day. The NTB, age, and BMI were standardized. NTB, dispositional need to belong; BMI, body mass index; *SE*, standard error; CI, confidence interval; LL, lower limit of 95% CI; UL, upper limit of 95% CI.

Table S3

The effect of social exclusion status on eating in participants with low and high dispositional need to belong with mixed effects modeling

| Low NTB |  |  |  |  |  |  |  |  |  |  | | |
| --- | --- | --- | --- | --- | --- | --- | --- | --- | --- | --- | --- | --- |
|  |  |  |  |  |  |  |  |  |  | 95% CI | | |
| Variable |  | *b* |  | *SE* |  | *t* |  | *p* |  | LL |  | UL |
| Social exclusion status |  | 0.09 |  | 0.04 |  | 2.27 |  | .026 |  | 0.01 |  | 0.16 |
| NTB_low |  | -0.03 |  | 0.06 |  | 0.51 |  | .611 |  | -0.14 |  | 0.08 |
| Social exclusion status  × NTB_low |  | -0.08 |  | 0.03 |  | 2.85 |  | .005 |  | -0.13 |  | -0.02 |
| Day |  | 0.39 |  | 0.03 |  | 14.26 |  | <.001 | | 0.33 |  | 0.44 |
| Age |  | 0.00 |  | 0.06 |  | 0.01 |  | .994 |  | -0.11 |  | 0.11 |
| BMI |  | 0.10 |  | 0.06 |  | 1.71 |  | .091 |  | -0.01 |  | 0.21 |
|  |  |  |  |  |  |  |  |  |  |  |  |  |
| High NTB |  |  |  |  |  |  |  |  |  |  |  |  |
|  |  |  |  |  |  |  |  |  |  | 95% CI | | |
| Variable |  | *b* |  | *SE* |  | *t* |  | *p* |  | LL |  | UL |
| Social exclusion status |  | -0.07 |  | 0.04 |  | 1.78 |  | .078 |  | -0.14 |  | 0.01 |
| NTB_high |  | -0.03 |  | 0.06 |  | 0.51 |  | .611 |  | -0.14 |  | 0.08 |
| Social exclusion status  × NTB_high |  | -0.08 |  | 0.03 |  | 2.85 |  | .005 |  | -0.13 |  | -0.02 |
| Day |  | 0.39 |  | 0.03 |  | 14.26 |  | <.001 | | 0.33 |  | 0.44 |
| Age |  | 0.00 |  | 0.06 |  | 0.01 |  | .994 |  | -0.11 |  | 0.12 |
| BMI |  | 0.10 |  | 0.06 |  | 1.71 |  | .091 |  | -0.02 |  | 0.22 |

*Note*. The dependent variable is the volume of food consumed with natural log transformation. Social exclusion status and day were dummy coded as -0.5 and 0.5 for inclusion and exclusion, respectively; -0.5 for the 1^st^ day and 0.5 for the 2^nd^ day. The NTB, age, and BMI were standardized. Low and high indicate that -1 SD and 1 SD are subtracted from the variable, respectively, to calculate the effect of social exclusion status in participants with low (-1 SD) and high (+1 SD) dispositional need to belong. The upper section of the table is for those with low dispositional need to belong and the lower section is for those with high dispositional need to belong. NTB, dispositional need to belong; BMI, body mass index; *SE*, standard error; CI, confidence interval; LL, lower limit of 95% CI; UL, upper limit of 95% CI.

Table S4

The effect of negative emotion on eating with mixed effects modeling

|  |  |  |  |  |  |  |  |  |  | 95% CI | | |
| --- | --- | --- | --- | --- | --- | --- | --- | --- | --- | --- | --- | --- |
| Variable |  | *b* |  | *SE* |  | *t* |  | *p* |  | LL |  | UL |
| Negative emotion change score |  | 0.01 |  | 0.04 |  | 0.14 |  | .888 |  | -0.07 |  | 0.08 |
| Negative emotion (Time 1) |  | 0.01 |  | 0.05 |  | 0.19 |  | .849 |  | -0.08 |  | 0.10 |
| Day |  | 0.39 |  | 0.03 |  | 13.45 |  | <.001 |  | 0.33 |  | 0.45 |
| Age |  | 0.00 |  | 0.06 |  | 0.06 |  | .953 |  | -0.11 |  | 0.12 |
| BMI |  | 0.10 |  | 0.06 |  | 1.69 |  | .094 |  | -0.02 |  | 0.21 |

*Note*. The dependent variable is the volume of food consumed with natural log transformation. Negative emotion change score was calculated by subtracting Time 1 from Time 2 negative emotion score. Day was dummy coded as -0.5 for the 1^st^ day and 0.5 for the 2^nd^ day. The age and BMI were standardized. BMI, body mass index; *SE*, standard error; CI, confidence interval; LL, lower limit of 95% CI; UL, upper limit of 95% CI.

Table S5

The effect of positive emotion on eating with mixed effects modeling

|  |  |  |  |  |  |  |  |  |  | 95% CI | | |
| --- | --- | --- | --- | --- | --- | --- | --- | --- | --- | --- | --- | --- |
| Variable |  | *b* |  | *SE* |  | *t* |  | *p* |  | LL |  | UL |
| Positive emotion change score |  | -0.05 |  | 0.04 |  | 1.07 |  | .287 |  | -0.13 |  | 0.04 |
| Positive emotion (Time 1) |  | -0.02 |  | 0.05 |  | 0.43 |  | .669 |  | -0.11 |  | 0.07 |
| Day |  | 0.39 |  | 0.03 |  | 13.14 |  | <.001 |  | 0.33 |  | 0.45 |
| Age |  | 0.00 |  | 0.06 |  | 0.08 |  | .940 |  | -0.11 |  | 0.12 |
| BMI |  | 0.10 |  | 0.06 |  | 1.69 |  | .095 |  | -0.02 |  | 0.21 |

*Note*. The dependent variable is the volume of food consumed with natural log transformation. Positive emotion change score was calculated by subtracting Time 1 from Time 2 positive emotion score. Day was dummy coded as -0.5 for the 1^st^ day and 0.5 for the 2^nd^ day. The age and BMI were standardized. BMI, body mass index; *SE*, standard error; CI, confidence interval; LL, lower limit of 95% CI; UL, upper limit of 95% CI.
